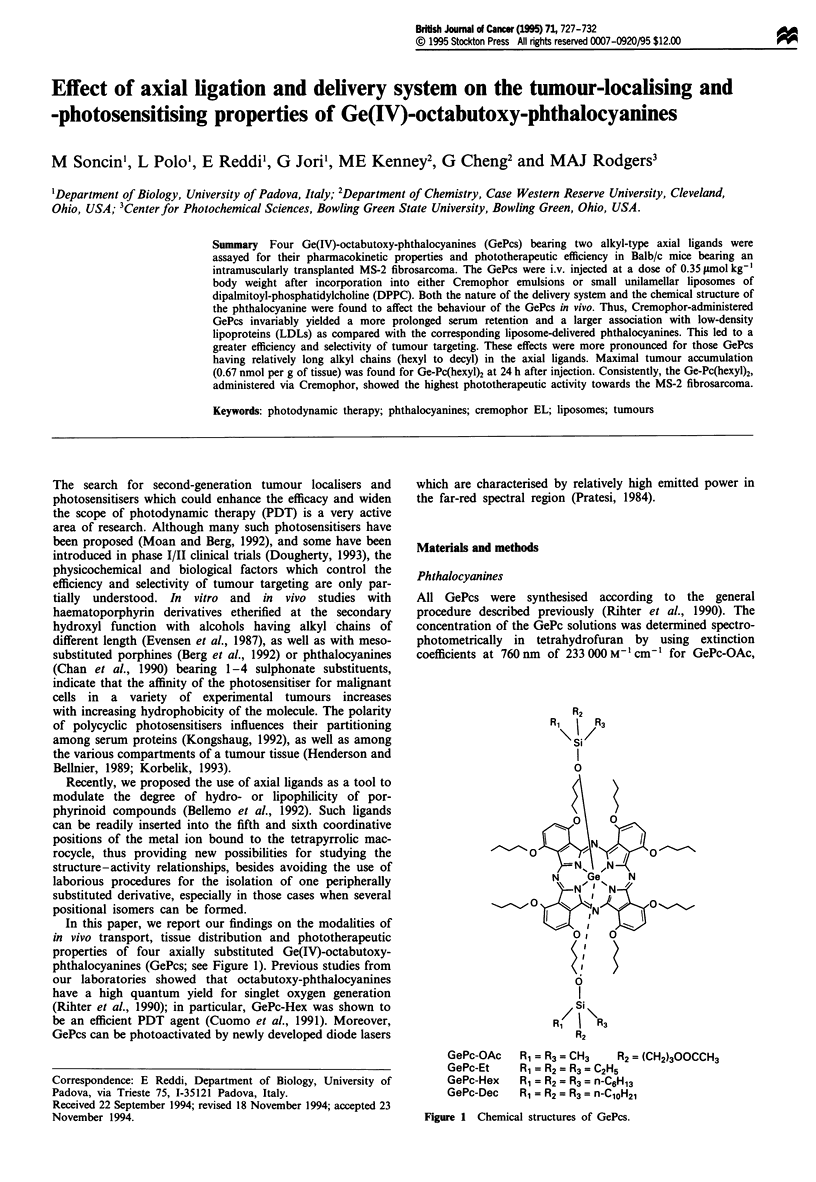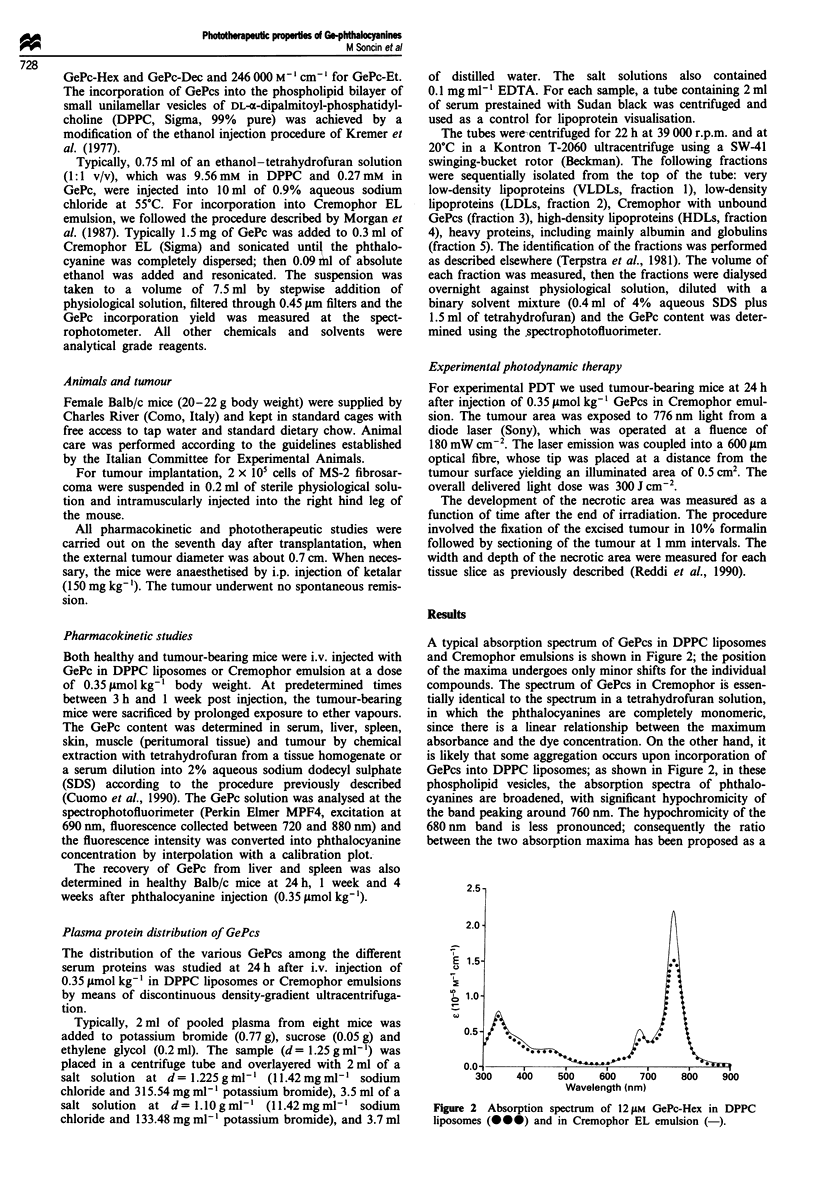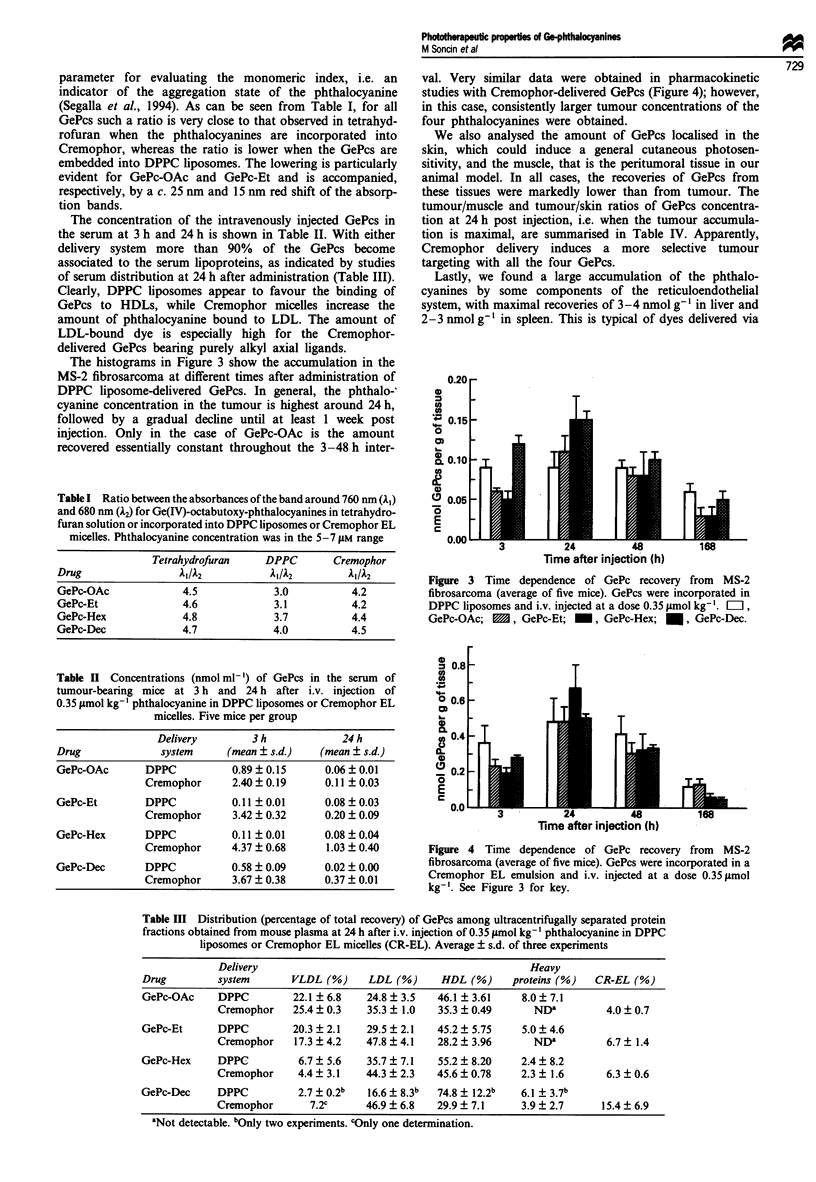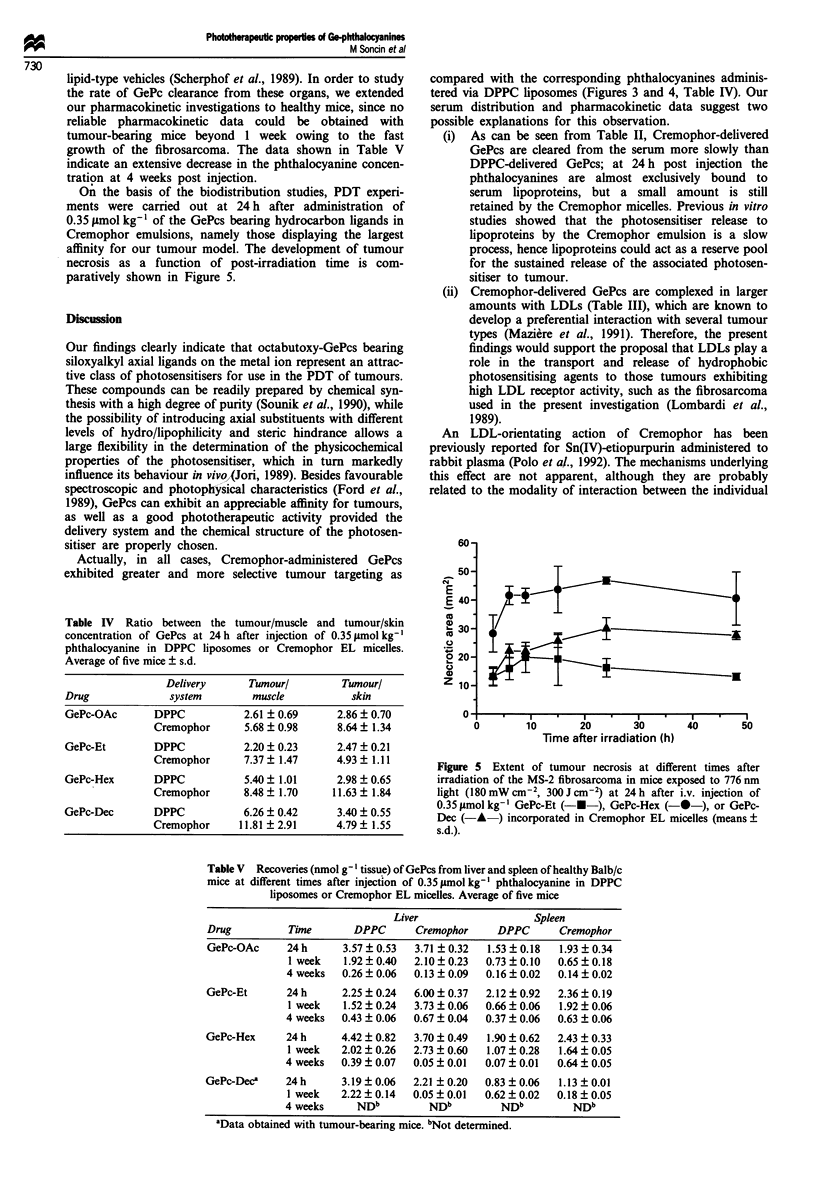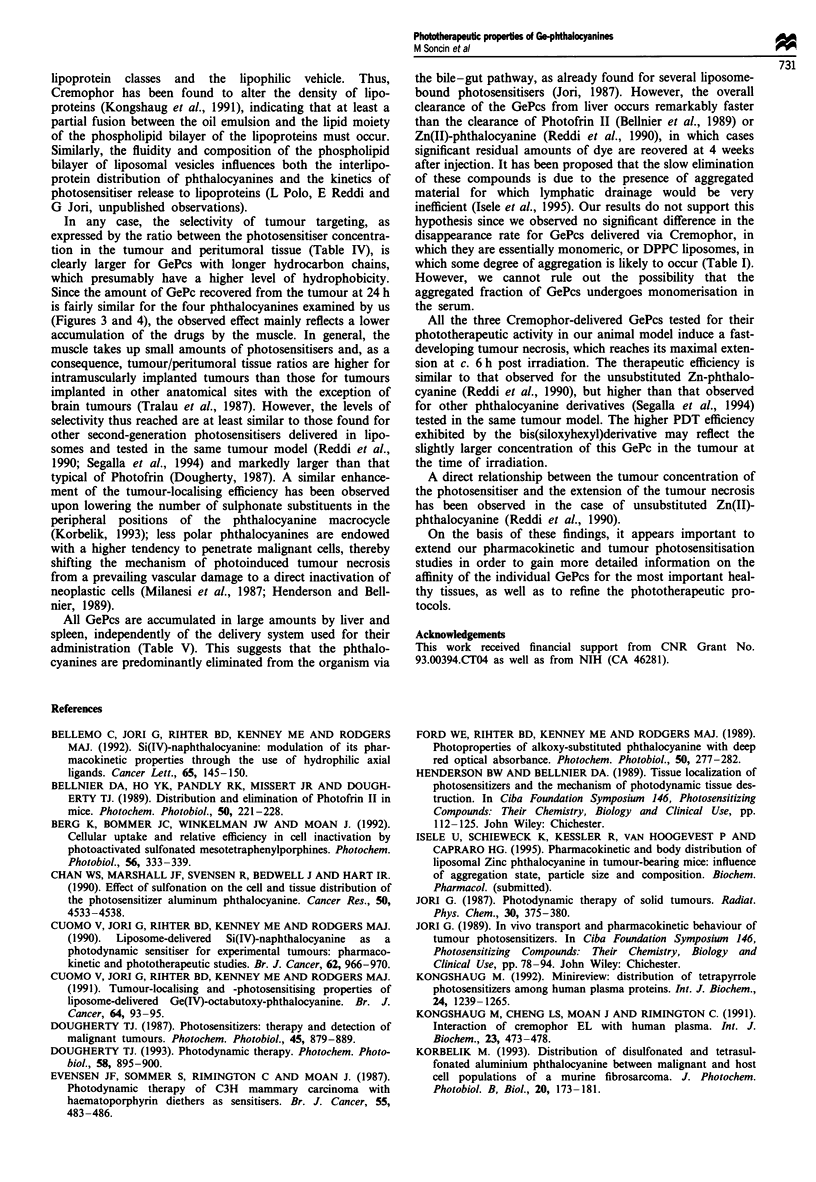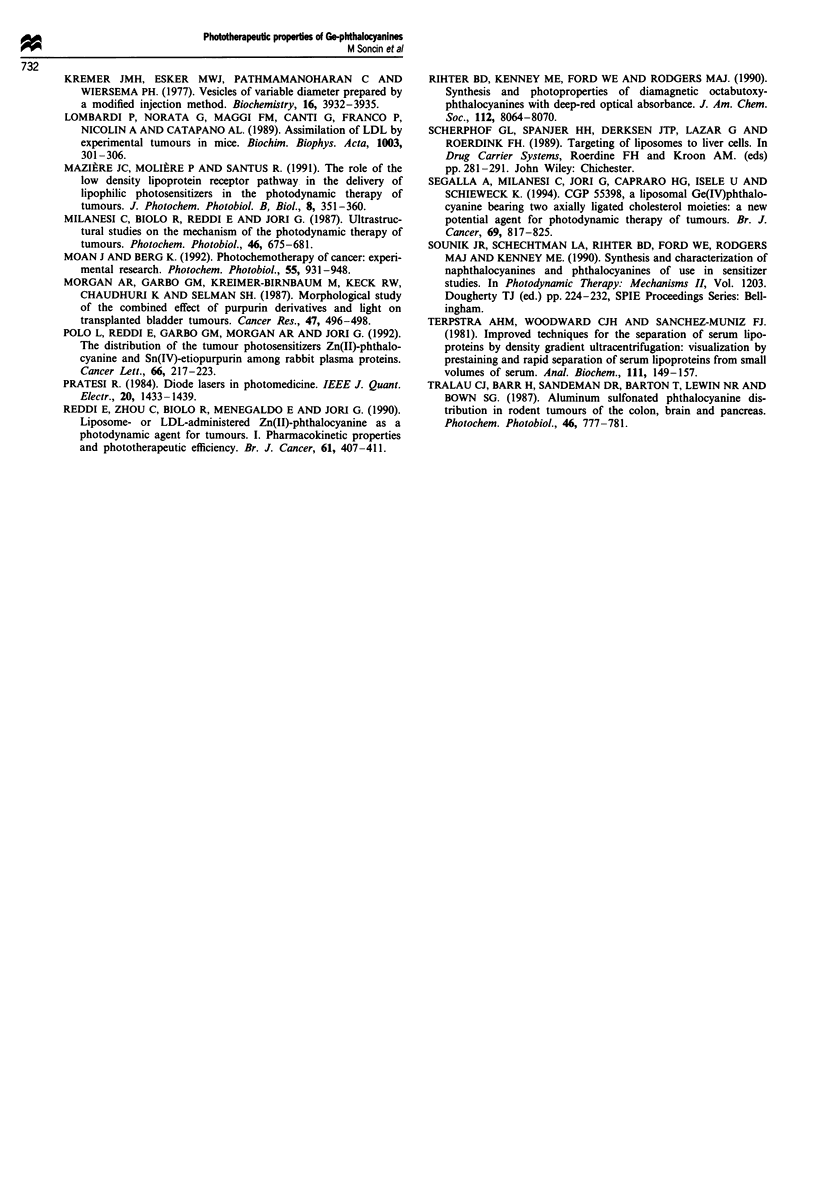# Effect of axial ligation and delivery system on the tumour-localising and -photosensitising properties of Ge(IV)-octabutoxy-phthalocyanines.

**DOI:** 10.1038/bjc.1995.142

**Published:** 1995-04

**Authors:** M. Soncin, L. Polo, E. Reddi, G. Jori, M. E. Kenney, G. Cheng, M. A. Rodgers

**Affiliations:** Department of Biology, University of Padova, Italy.

## Abstract

Four Ge(IV)-octabutoxy-phthalocyanines (GePcs) bearing two alkyl-type axial ligands were assayed for their pharmacokinetic properties and phototherapeutic efficiency in Balb/c mice bearing an intramuscularly transplanted MS-2 fibrosarcoma. The GePcs were i.v. injected at a dose of 0.35 mumol kg-1 body weight after incorporation into either Cremophor emulsions or small unilamellar liposomes of dipalmitoyl-phosphatidylcholine (DPPC). Both the nature of the delivery system and the chemical structure of the phthalocyanine were found to affect the behaviour of the GePcs in vivo. Thus, Cremophor-administered GePcs invariably yielded a more prolonged serum retention and a larger association with low-density lipoproteins (LDLs) as compared with the corresponding liposome-delivered phthalocyanines. This led to a greater efficiency and selectivity of tumour targeting. These effects were more pronounced for those GePcs having relatively long alkyl chains (hexyl to decyl) in the axial ligands. Maximal tumour accumulation (0.67 nmol per g of tissue) was found for Ge-Pc(hexyl)2 at 24 h after injection. Consistently, the Ge-Pc(hexyl)2, administered via Cremophor, showed the highest phototherapeutic activity towards MS-2 fibrosarcoma.


British Journal of Cancer (1995) 71, 727-732
© 1995 Stockton Press All rights reserved 0007-0920/95 $12.00

### Effect of axial ligation and delivery system on the tumour-localising and -photosensitising properties of Ge(IV)-octabutoxy-phthalocyanines

M Soncin[1], L Polo[1], E Reddi[1], G Jori[1], ME Kenney[2], G Cheng[2] and MAJ Rodgers[3]

[1]*Department of Biology, University of Padova, Italy;* [2]*Department of Chemistry, Case Western Reserve University, Cleveland, Ohio, USA;* [3]*Center for Photochemical Sciences, Bowling Green State University, Bowling Green, Ohio, USA.*

**Summary** Four Ge(IV)-octabutoxy-phthalocyanines (GePcs) bearing two alkyl-type axial ligands were assayed for their pharmacokinetic properties and phototherapeutic efficiency in Balb/c mice bearing an intramuscularly transplanted MS-2 fibrosarcoma. The GePcs were i.v. injected at a dose of 0.35 $\mu$mol $kg^{-1}$ body weight after incorporation into either Cremophor emulsions or small unilamellar liposomes of dipalmitoyl-phosphatidylcholine (DPPC). Both the nature of the delivery system and the chemical structure of the phthalocyanine were found to affect the behaviour of the GePcs *in vivo*. Thus, Cremophor-administered GePcs invariably yielded a more prolonged serum retention and a larger association with low-density lipoproteins (LDLs) as compared with the corresponding liposome-delivered phthalocyanines. This led to a greater efficiency and selectivity of tumour targeting. These effects were more pronounced for those GePcs having relatively long alkyl chains (hexyl to decyl) in the axial ligands. Maximal tumour accumulation (0.67 nmol per g of tissue) was found for Ge-Pc(hexyl)$_2$ at 24 h after injection. Consistently, the Ge-Pc(hexyl)$_2$, administered via Cremophor, showed the highest phototherapeutic activity towards the MS-2 fibrosarcoma.

**Keywords**: photodynamic therapy; phthalocyanines; cremophor EL; liposomes; tumours

The search for second-generation tumour localisers and photosensitisers which could enhance the efficacy and widen the scope of photodynamic therapy (PDT) is a very active area of research. Although many such photosensitisers have been proposed (Moan and Berg, 1992), and some have been introduced in phase I/II clinical trials (Dougherty, 1993), the physicochemical and biological factors which control the efficiency and selectivity of tumour targeting are only partially understood. *In vitro* and *in vivo* studies with haematoporphyrin derivatives etherified at the secondary hydroxyl function with alcohols having alkyl chains of different length (Evensen *et al.*, 1987), as well as with meso-substituted porphines (Berg *et al.*, 1992) or phthalocyanines (Chan *et al.*, 1990) bearing 1-4 sulphonate substituents, indicate that the affinity of the photosensitiser for malignant cells in a variety of experimental tumours increases with increasing hydrophobicity of the molecule. The polarity of polycyclic photosensitisers influences their partitioning among serum proteins (Kongshaug, 1992), as well as among the various compartments of a tumour tissue (Henderson and Bellnier, 1989; Korbelik, 1993).

Recently, we proposed the use of axial ligands as a tool to modulate the degree of hydro- or lipophilicity of porphyrinoid compounds (Bellemo *et al.*, 1992). Such ligands can be readily inserted into the fifth and sixth coordinative positions of the metal ion bound to the tetrapyrrolic macrocycle, thus providing new possibilities for studying the structure-activity relationships, besides avoiding the use of laborious procedures for the isolation of one peripherally substituted derivative, especially in those cases when several positional isomers can be formed.

In this paper, we report our findings on the modalities of *in vivo* transport, tissue distribution and phototherapeutic properties of four axially substituted Ge(IV)-octabutoxy-phthalocyanines (GePcs; see Figure 1). Previous studies from our laboratories showed that octabutoxy-phthalocyanines have a high quantum yield for singlet oxygen generation (Rihter *et al.*, 1990); in particular, GePc-Hex was shown to be an efficient PDT agent (Cuomo *et al.*, 1991). Moreover, GePcs can be photoactivated by newly developed diode lasers which are characterised by relatively high emitted power in the far-red spectral region (Pratesi, 1984).

### Materials and methods

### *Phthalocyanines*

All GePcs were synthesised according to the general procedure described previously (Rihter *et al.*, 1990). The concentration of the GePc solutions was determined spectrophotometrically in tetrahydrofuran by using extinction coefficients at 760 nm of 233 000 $M^{-1}$ $cm^{-1}$ for GePc-OAc,

| | | |
|---|---|---|
| GePc-OAc | $R_1 = R_3 = CH_3$ | $R_2 = (CH_2)_3OOCCH_3$ |
| GePc-Et | $R_1 = R_2 = R_3 = C_2H_5$ | |
| GePc-Hex | $R_1 = R_2 = R_3 = n\text{-}C_6H_{13}$ | |
| GePc-Dec | $R_1 = R_2 = R_3 = n\text{-}C_{10}H_{21}$ | |

**Figure 1** Chemical structures of GePcs.

Correspondence: E Reddi, Department of Biology, University of Padova, via Trieste 75, I-35121 Padova, Italy.
Received 22 September 1994; revised 18 November 1994; accepted 23 November 1994.

GePc-Hex and GePc-Dec and 246 000 $M^{-1}$ $cm^{-1}$ for GePc-Et. The incorporation of GePcs into the phospholipid bilayer of small unilamellar vesicles of DL-α-dipalmitoyl-phosphatidylcholine (DPPC, Sigma, 99% pure) was achieved by a modification of the ethanol injection procedure of Kremer *et al.* (1977).

Typically, 0.75 ml of an ethanol–tetrahydrofuran solution (1:1 v/v), which was 9.56 mM in DPPC and 0.27 mM in GePc, were injected into 10 ml of 0.9% aqueous sodium chloride at 55°C. For incorporation into Cremophor EL emulsion, we followed the procedure described by Morgan *et al.* (1987). Typically 1.5 mg of GePc was added to 0.3 ml of Cremophor EL (Sigma) and sonicated until the phthalocyanine was completely dispersed; then 0.09 ml of absolute ethanol was added and resonicated. The suspension was taken to a volume of 7.5 ml by stepwise addition of physiological solution, filtered through 0.45 μm filters and the GePc incorporation yield was measured at the spectrophotometer. All other chemicals and solvents were analytical grade reagents.

### *Animals and tumour*

Female Balb/c mice (20–22 g body weight) were supplied by Charles River (Como, Italy) and kept in standard cages with free access to tap water and standard dietary chow. Animal care was performed according to the guidelines established by the Italian Committee for Experimental Animals.

For tumour implantation, $2 \times 10^5$ cells of MS-2 fibrosarcoma were suspended in 0.2 ml of sterile physiological solution and intramuscularly injected into the right hind leg of the mouse.

All pharmacokinetic and phototherapeutic studies were carried out on the seventh day after transplantation, when the external tumour diameter was about 0.7 cm. When necessary, the mice were anaesthetised by i.p. injection of ketalar (150 mg $kg^{-1}$). The tumour underwent no spontaneous remission.

### *Pharmacokinetic studies*

Both healthy and tumour-bearing mice were i.v. injected with GePc in DPPC liposomes or Cremophor emulsion at a dose of 0.35 μmol $kg^{-1}$ body weight. At predetermined times between 3 h and 1 week post injection, the tumour-bearing mice were sacrificed by prolonged exposure to ether vapours. The GePc content was determined in serum, liver, spleen, skin, muscle (peritumoral tissue) and tumour by chemical extraction with tetrahydrofuran from a tissue homogenate or a serum dilution into 2% aqueous sodium dodecyl sulphate (SDS) according to the procedure previously described (Cuomo *et al.*, 1990). The GePc solution was analysed at the spectrophotofluorimeter (Perkin Elmer MPF4, excitation at 690 nm, fluorescence collected between 720 and 880 nm) and the fluorescence intensity was converted into phthalocyanine concentration by interpolation with a calibration plot.

The recovery of GePc from liver and spleen was also determined in healthy Balb/c mice at 24 h, 1 week and 4 weeks after phthalocyanine injection (0.35 μmol $kg^{-1}$).

### *Plasma protein distribution of GePcs*

The distribution of the various GePcs among the different serum proteins was studied at 24 h after i.v. injection of 0.35 μmol $kg^{-1}$ in DPPC liposomes or Cremophor emulsions by means of discontinuous density-gradient ultracentrifugation.

Typically, 2 ml of pooled plasma from eight mice was added to potassium bromide (0.77 g), sucrose (0.05 g) and ethylene glycol (0.2 ml). The sample ($d = 1.25$ g $ml^{-1}$) was placed in a centrifuge tube and overlayered with 2 ml of a salt solution at $d = 1.225$ g $ml^{-1}$ (11.42 mg $ml^{-1}$ sodium chloride and 315.54 mg $ml^{-1}$ potassium bromide), 3.5 ml of a salt solution at $d = 1.10$ g $ml^{-1}$ (11.42 mg $ml^{-1}$ sodium chloride and 133.48 mg $ml^{-1}$ potassium bromide), and 3.7 ml of distilled water. The salt solutions also contained 0.1 mg $ml^{-1}$ EDTA. For each sample, a tube containing 2 ml of serum prestained with Sudan black was centrifuged and used as a control for lipoprotein visualisation.

The tubes were centrifuged for 22 h at 39 000 r.p.m. and at 20°C in a Kontron T-2060 ultracentrifuge using a SW-41 swinging-bucket rotor (Beckman). The following fractions were sequentially isolated from the top of the tube: very low-density lipoproteins (VLDLs, fraction 1), low-density lipoproteins (LDLs, fraction 2), Cremophor with unbound GePcs (fraction 3), high-density lipoproteins (HDLs, fraction 4), heavy proteins, including mainly albumin and globulins (fraction 5). The identification of the fractions was performed as described elsewhere (Terpstra *et al.*, 1981). The volume of each fraction was measured, then the fractions were dialysed overnight against physiological solution, diluted with a binary solvent mixture (0.4 ml of 4% aqueous SDS plus 1.5 ml of tetrahydrofuran) and the GePc content was determined using the spectrophotofluorimeter.

### *Experimental photodynamic therapy*

For experimental PDT we used tumour-bearing mice at 24 h after injection of 0.35 μmol $kg^{-1}$ GePcs in Cremophor emulsion. The tumour area was exposed to 776 nm light from a diode laser (Sony), which was operated at a fluence of 180 mW $cm^{-2}$. The laser emission was coupled into a 600 μm optical fibre, whose tip was placed at a distance from the tumour surface yielding an illuminated area of 0.5 $cm^2$. The overall delivered light dose was 300 J $cm^{-2}$.

The development of the necrotic area was measured as a function of time after the end of irradiation. The procedure involved the fixation of the excised tumour in 10% formalin followed by sectioning of the tumour at 1 mm intervals. The width and depth of the necrotic area were measured for each tissue slice as previously described (Reddi *et al.*, 1990).

### Results

A typical absorption spectrum of GePcs in DPPC liposomes and Cremophor emulsions is shown in Figure 2; the position of the maxima undergoes only minor shifts for the individual compounds. The spectrum of GePcs in Cremophor is essentially identical to the spectrum in a tetrahydrofuran solution, in which the phthalocyanines are completely monomeric, since there is a linear relationship between the maximum absorbance and the dye concentration. On the other hand, it is likely that some aggregation occurs upon incorporation of GePcs into DPPC liposomes; as shown in Figure 2, in these phospholipid vesicles, the absorption spectra of phthalocyanines are broadened, with significant hypochromicity of the band peaking around 760 nm. The hypochromicity of the 680 nm band is less pronounced; consequently the ratio between the two absorption maxima has been proposed as a

**Figure 2** Absorption spectrum of 12 μM GePc-Hex in DPPC liposomes (●●●) and in Cremophor EL emulsion (—).

parameter for evaluating the monomeric index, i.e. an indicator of the aggregation state of the phthalocyanine (Segalla *et al.*, 1994). As can be seen from Table I, for all GePcs such a ratio is very close to that observed in tetrahydrofuran when the phthalocyanines are incorporated into Cremophor, whereas the ratio is lower when the GePcs are embedded into DPPC liposomes. The lowering is particularly evident for GePc-OAc and GePc-Et and is accompanied, respectively, by a *c.* 25 nm and 15 nm red shift of the absorption bands.

The concentration of the intravenously injected GePcs in the serum at 3 h and 24 h is shown in Table II. With either delivery system more than 90% of the GePcs become associated to the serum lipoproteins, as indicated by studies of serum distribution at 24 h after administration (Table III). Clearly, DPPC liposomes appear to favour the binding of GePcs to HDLs, while Cremophor micelles increase the amount of phthalocyanine bound to LDL. The amount of LDL-bound dye is especially high for the Cremophor-delivered GePcs bearing purely alkyl axial ligands.

The histograms in Figure 3 show the accumulation in the MS-2 fibrosarcoma at different times after administration of DPPC liposome-delivered GePcs. In general, the phthalocyanine concentration in the tumour is highest around 24 h, followed by a gradual decline until at least 1 week post injection. Only in the case of GePc-OAc is the amount recovered essentially constant throughout the 3–48 h interval. Very similar data were obtained in pharmacokinetic studies with Cremophor-delivered GePcs (Figure 4); however, in this case, consistently larger tumour concentrations of the four phthalocyanines were obtained.

We also analysed the amount of GePcs localised in the skin, which could induce a general cutaneous photosensitivity, and the muscle, that is the peritumoral tissue in our animal model. In all cases, the recoveries of GePcs from these tissues were markedly lower than from tumour. The tumour/muscle and tumour/skin ratios of GePcs concentration at 24 h post injection, i.e. when the tumour accumulation is maximal, are summarised in Table IV. Apparently, Cremophor delivery induces a more selective tumour targeting with all the four GePcs.

Lastly, we found a large accumulation of the phthalocyanines by some components of the reticuloendothelial system, with maximal recoveries of 3–4 nmol $g^{-1}$ in liver and 2–3 nmol $g^{-1}$ in spleen. This is typical of dyes delivered via

**Table I** Ratio between the absorbances of the band around 760 nm ($\lambda_1$) and 680 nm ($\lambda_2$) for Ge(IV)-octabutoxy-phthalocyanines in tetrahydrofuran solution or incorporated into DPPC liposomes or Cremophor EL micelles. Phthalocyanine concentration was in the 5–7 μM range

| Drug | *Tetrahydrofuran* $\lambda_1/\lambda_2$ | *DPPC* $\lambda_1/\lambda_2$ | *Cremophor* $\lambda_1/\lambda_2$ |
|---|---|---|---|
| GePc-OAc | 4.5 | 3.0 | 4.2 |
| GePc-Et | 4.6 | 3.1 | 4.2 |
| GePc-Hex | 4.8 | 3.7 | 4.4 |
| GePc-Dec | 4.7 | 4.0 | 4.5 |

**Table II** Concentrations (nmol $ml^{-1}$) of GePcs in the serum of tumour-bearing mice at 3 h and 24 h after i.v. injection of 0.35 μmol $kg^{-1}$ phthalocyanine in DPPC liposomes or Cremophor EL micelles. Five mice per group

| *Drug* | *Delivery system* | *3 h (mean ± s.d.)* | *24 h (mean ± s.d.)* |
|---|---|---|---|
| GePc-OAc | DPPC | 0.89 ± 0.15 | 0.06 ± 0.01 |
| | Cremophor | 2.40 ± 0.19 | 0.11 ± 0.03 |
| GePc-Et | DPPC | 0.11 ± 0.01 | 0.08 ± 0.03 |
| | Cremophor | 3.42 ± 0.32 | 0.20 ± 0.09 |
| GePc-Hex | DPPC | 0.11 ± 0.01 | 0.08 ± 0.04 |
| | Cremophor | 4.37 ± 0.68 | 1.03 ± 0.40 |
| GePc-Dec | DPPC | 0.58 ± 0.09 | 0.02 ± 0.00 |
| | Cremophor | 3.67 ± 0.38 | 0.37 ± 0.01 |

**Figure 3** Time dependence of GePc recovery from MS-2 fibrosarcoma (average of five mice). GePcs were incorporated in DPPC liposomes and i.v. injected at a dose 0.35 μmol $kg^{-1}$. ☐, GePc-OAc; ▨, GePc-Et; ■, GePc-Hex; ▩, GePc-Dec.

**Figure 4** Time dependence of GePc recovery from MS-2 fibrosarcoma (average of five mice). GePcs were incorporated in a Cremophor EL emulsion and i.v. injected at a dose 0.35 μmol $kg^{-1}$. See Figure 3 for key.

**Table III** Distribution (percentage of total recovery) of GePcs among ultracentrifugally separated protein fractions obtained from mouse plasma at 24 h after i.v. injection of 0.35 μmol $kg^{-1}$ phthalocyanine in DPPC liposomes or Cremophor EL micelles (CR-EL). Average ± s.d. of three experiments

| *Drug* | *Delivery system* | *VLDL (%)* | *LDL (%)* | *HDL (%)* | *Heavy proteins (%)* | *CR-EL (%)* |
|---|---|---|---|---|---|---|
| GePc-OAc | DPPC | 22.1 ± 6.8 | 24.8 ± 3.5 | 46.1 ± 3.61 | 8.0 ± 7.1 | |
| | Cremophor | 25.4 ± 0.3 | 35.3 ± 1.0 | 35.3 ± 0.49 | ND[a] | 4.0 ± 0.7 |
| GePc-Et | DPPC | 20.3 ± 2.1 | 29.5 ± 2.1 | 45.2 ± 5.75 | 5.0 ± 4.6 | |
| | Cremophor | 17.3 ± 4.2 | 47.8 ± 4.1 | 28.2 ± 3.96 | ND[a] | 6.7 ± 1.4 |
| GePc-Hex | DPPC | 6.7 ± 5.6 | 35.7 ± 7.1 | 55.2 ± 8.20 | 2.4 ± 8.2 | |
| | Cremophor | 4.4 ± 3.1 | 44.3 ± 2.3 | 45.6 ± 0.78 | 2.3 ± 1.6 | 6.3 ± 0.6 |
| GePc-Dec | DPPC | 2.7 ± 0.2[b] | 16.6 ± 8.3[b] | 74.8 ± 12.2[b] | 6.1 ± 3.7[b] | |
| | Cremophor | 7.2[c] | 46.9 ± 6.8 | 29.9 ± 7.1 | 3.9 ± 2.7 | 15.4 ± 6.9 |

[a]Not detectable. [b]Only two experiments. [c]Only one determination.

lipid-type vehicles (Scherphof et al., 1989). In order to study the rate of GePc clearance from these organs, we extended our pharmacokinetic investigations to healthy mice, since no reliable pharmacokinetic data could be obtained with tumour-bearing mice beyond 1 week owing to the fast growth of the fibrosarcoma. The data shown in Table V indicate an extensive decrease in the phthalocyanine concentration at 4 weeks post injection.

On the basis of the biodistribution studies, PDT experiments were carried out at 24 h after administration of 0.35 $\mu$mol $kg^{-1}$ of the GePcs bearing hydrocarbon ligands in Cremophor emulsions, namely those displaying the largest affinity for our tumour model. The development of tumour necrosis as a function of post-irradiation time is comparatively shown in Figure 5.

### Discussion

Our findings clearly indicate that octabutoxy-GePcs bearing siloxyalkyl axial ligands on the metal ion represent an attractive class of photosensitisers for use in the PDT of tumours. These compounds can be readily prepared by chemical synthesis with a high degree of purity (Sounik et al., 1990), while the possibility of introducing axial substituents with different levels of hydro/lipophilicity and steric hindrance allows a large flexibility in the determination of the physicochemical properties of the photosensitiser, which in turn markedly influence its behaviour in vivo (Jori, 1989). Besides favourable spectroscopic and photophysical characteristics (Ford et al., 1989), GePcs can exhibit an appreciable affinity for tumours, as well as a good phototherapeutic activity provided the delivery system and the chemical structure of the photosensitiser are properly chosen.

Actually, in all cases, Cremophor-administered GePcs exhibited greater and more selective tumour targeting as compared with the corresponding phthalocyanines administered via DPPC liposomes (Figures 3 and 4, Table IV). Our serum distribution and pharmacokinetic data suggest two possible explanations for this observation.

(i) As can be seen from Table II, Cremophor-delivered GePcs are cleared from the serum more slowly than DPPC-delivered GePcs; at 24 h post injection the phthalocyanines are almost exclusively bound to serum lipoproteins, but a small amount is still retained by the Cremophor micelles. Previous in vitro studies showed that the photosensitiser release to lipoproteins by the Cremophor emulsion is a slow process, hence lipoproteins could act as a reserve pool for the sustained release of the associated photosensitiser to tumour.

(ii) Cremophor-delivered GePcs are complexed in larger amounts with LDLs (Table III), which are known to develop a preferential interaction with several tumour types (Mazière et al., 1991). Therefore, the present findings would support the proposal that LDLs play a role in the transport and release of hydrophobic photosensitising agents to those tumours exhibiting high LDL receptor activity, such as the fibrosarcoma used in the present investigation (Lombardi et al., 1989).

An LDL-orientating action of Cremophor has been previously reported for Sn(IV)-etiopurpurin administered to rabbit plasma (Polo et al., 1992). The mechanisms underlying this effect are not apparent, although they are probably related to the modality of interaction between the individual

**Table IV** Ratio between the tumour/muscle and tumour/skin concentration of GePcs at 24 h after injection of 0.35 $\mu$mol $kg^{-1}$ phthalocyanine in DPPC liposomes or Cremophor EL micelles. Average of five mice ± s.d.

| Drug | Delivery system | Tumour/ muscle | Tumour/ skin |
|---|---|---|---|
| GePc-OAc | DPPC | 2.61 ± 0.69 | 2.86 ± 0.70 |
| | Cremophor | 5.68 ± 0.98 | 8.64 ± 1.34 |
| GePc-Et | DPPC | 2.20 ± 0.23 | 2.47 ± 0.21 |
| | Cremophor | 7.37 ± 1.47 | 4.93 ± 1.11 |
| GePc-Hex | DPPC | 5.40 ± 1.01 | 2.98 ± 0.65 |
| | Cremophor | 8.48 ± 1.70 | 11.63 ± 1.84 |
| GePc-Dec | DPPC | 6.26 ± 0.42 | 3.40 ± 0.55 |
| | Cremophor | 11.81 ± 2.91 | 4.79 ± 1.55 |

**Figure 5** Extent of tumour necrosis at different times after irradiation of the MS-2 fibrosarcoma in mice exposed to 776 nm light (180 mW $cm^{-2}$, 300 J $cm^{-2}$) at 24 h after i.v. injection of 0.35 $\mu$mol $kg^{-1}$ GePc-Et (—■—), GePc-Hex (—●—), or GePc-Dec (—▲—) incorporated in Cremophor EL micelles (means ± s.d.).

**Table V** Recoveries (nmol $g^{-1}$ tissue) of GePcs from liver and spleen of healthy Balb/c mice at different times after injection of 0.35 $\mu$mol $kg^{-1}$ phthalocyanine in DPPC liposomes or Cremophor EL micelles. Average of five mice

| Drug | Time | Liver DPPC | Liver Cremophor | Spleen DPPC | Spleen Cremophor |
|---|---|---|---|---|---|
| GePc-OAc | 24 h | 3.57 ± 0.53 | 3.71 ± 0.32 | 1.53 ± 0.18 | 1.93 ± 0.34 |
| | 1 week | 1.92 ± 0.40 | 2.10 ± 0.23 | 0.73 ± 0.10 | 0.65 ± 0.18 |
| | 4 weeks | 0.26 ± 0.06 | 0.13 ± 0.09 | 0.16 ± 0.02 | 0.14 ± 0.02 |
| GePc-Et | 24 h | 2.25 ± 0.24 | 6.00 ± 0.37 | 2.12 ± 0.92 | 2.36 ± 0.19 |
| | 1 week | 1.52 ± 0.24 | 3.73 ± 0.06 | 0.66 ± 0.06 | 1.92 ± 0.06 |
| | 4 weeks | 0.43 ± 0.06 | 0.67 ± 0.04 | 0.37 ± 0.06 | 0.63 ± 0.06 |
| GePc-Hex | 24 h | 4.42 ± 0.82 | 3.70 ± 0.49 | 1.90 ± 0.62 | 2.43 ± 0.33 |
| | 1 week | 2.02 ± 0.26 | 2.73 ± 0.60 | 1.07 ± 0.28 | 1.64 ± 0.05 |
| | 4 weeks | 0.39 ± 0.07 | 0.05 ± 0.01 | 0.07 ± 0.01 | 0.64 ± 0.05 |
| GePc-Dec[a] | 24 h | 3.19 ± 0.06 | 2.21 ± 0.20 | 0.83 ± 0.06 | 1.13 ± 0.01 |
| | 1 week | 2.22 ± 0.14 | 0.05 ± 0.01 | 0.62 ± 0.02 | 0.18 ± 0.05 |
| | 4 weeks | ND[b] | ND[b] | ND[b] | ND[b] |

[a]Data obtained with tumour-bearing mice. [b]Not determined.

lipoprotein classes and the lipophilic vehicle. Thus, Cremophor has been found to alter the density of lipoproteins (Kongshaug et al., 1991), indicating that at least a partial fusion between the oil emulsion and the lipid moiety of the phospholipid bilayer of the lipoproteins must occur. Similarly, the fluidity and composition of the phospholipid bilayer of liposomal vesicles influences both the interlipoprotein distribution of phthalocyanines and the kinetics of photosensitiser release to lipoproteins (L Polo, E Reddi and G Jori, unpublished observations).

In any case, the selectivity of tumour targeting, as expressed by the ratio between the photosensitiser concentration in the tumour and peritumoral tissue (Table IV), is clearly larger for GePcs with longer hydrocarbon chains, which presumably have a higher level of hydrophobicity. Since the amount of GePc recovered from the tumour at 24 h is fairly similar for the four phthalocyanines examined by us (Figures 3 and 4), the observed effect mainly reflects a lower accumulation of the drugs by the muscle. In general, the muscle takes up small amounts of photosensitisers and, as a consequence, tumour/peritumoral tissue ratios are higher for intramuscularly implanted tumours than those for tumours implanted in other anatomical sites with the exception of brain tumours (Tralau et al., 1987). However, the levels of selectivity thus reached are at least similar to those found for other second-generation photosensitisers delivered in liposomes and tested in the same tumour model (Reddi et al., 1990; Segalla et al., 1994) and markedly larger than that typical of Photofrin (Dougherty, 1987). A similar enhancement of the tumour-localising efficiency has been observed upon lowering the number of sulphonate substituents in the peripheral positions of the phthalocyanine macrocycle (Korbelik, 1993); less polar phthalocyanines are endowed with a higher tendency to penetrate malignant cells, thereby shifting the mechanism of photoinduced tumour necrosis from a prevailing vascular damage to a direct inactivation of neoplastic cells (Milanesi et al., 1987; Henderson and Bellnier, 1989).

All GePcs are accumulated in large amounts by liver and spleen, independently of the delivery system used for their administration (Table V). This suggests that the phthalocyanines are predominantly eliminated from the organism via the bile–gut pathway, as already found for several liposome-bound photosensitisers (Jori, 1987). However, the overall clearance of the GePcs from liver occurs remarkably faster than the clearance of Photofrin II (Bellnier et al., 1989) or Zn(II)-phthalocyanine (Reddi et al., 1990), in which cases significant residual amounts of dye are reovered at 4 weeks after injection. It has been proposed that the slow elimination of these compounds is due to the presence of aggregated material for which lymphatic drainage would be very inefficient (Isele et al., 1995). Our results do not support this hypothesis since we observed no significant difference in the disappearance rate for GePcs delivered via Cremophor, in which they are essentially monomeric, or DPPC liposomes, in which some degree of aggregation is likely to occur (Table I). However, we cannot rule out the possibility that the aggregated fraction of GePcs undergoes monomerisation in the serum.

All the three Cremophor-delivered GePcs tested for their phototherapeutic activity in our animal model induce a fast-developing tumour necrosis, which reaches its maximal extension at c. 6 h post irradiation. The therapeutic efficiency is similar to that observed for the unsubstituted Zn-phthalocyanine (Reddi et al., 1990), but higher than that observed for other phthalocyanine derivatives (Segalla et al., 1994) tested in the same tumour model. The higher PDT efficiency exhibited by the bis(siloxyhexyl)derivative may reflect the slightly larger concentration of this GePc in the tumour at the time of irradiation.

A direct relationship between the tumour concentration of the photosensitiser and the extension of the tumour necrosis has been observed in the case of unsubstituted Zn(II)-phthalocyanine (Reddi et al., 1990).

On the basis of these findings, it appears important to extend our pharmacokinetic and tumour photosensitisation studies in order to gain more detailed information on the affinity of the individual GePcs for the most important healthy tissues, as well as to refine the phototherapeutic protocols.

**Acknowledgements**
This work received financial support from CNR Grant No. 93.00394.CT04 as well as from NIH (CA 46281).

### References

BELLEMO C, JORI G, RIHTER BD, KENNEY ME AND RODGERS MAJ. (1992). Si(IV)-naphthalocyanine: modulation of its pharmacokinetic properties through the use of hydrophilic axial ligands. *Cancer Lett.*, **65**, 145–150.

BELLNIER DA, HO YK, PANDLY RK, MISSERT JR AND DOUGHERTY TJ. (1989). Distribution and elimination of Photofrin II in mice. *Photochem. Photobiol.*, **50**, 221–228.

BERG K, BOMMER JC, WINKELMAN JW AND MOAN J. (1992). Cellular uptake and relative efficiency in cell inactivation by photoactivated sulfonated mesotetraphenylporphines. *Photochem. Photobiol.*, **56**, 333–339.

CHAN WS, MARSHALL JF, SVENSEN R, BEDWELL J AND HART IR. (1990). Effect of sulfonation on the cell and tissue distribution of the photosensitizer aluminum phthalocyanine. *Cancer Res.*, **50**, 4533–4538.

CUOMO V, JORI G, RIHTER BD, KENNEY ME AND RODGERS MAJ. (1990). Liposome-delivered Si(IV)-naphthalocyanine as a photodynamic sensitiser for experimental tumours: pharmacokinetic and phototherapeutic studies. *Br. J. Cancer*, **62**, 966–970.

CUOMO V, JORI G, RIHTER BD, KENNEY ME AND RODGERS MAJ. (1991). Tumour-localising and -photosensitising properties of liposome-delivered Ge(IV)-octabutoxy-phthalocyanine. *Br. J. Cancer*, **64**, 93–95.

DOUGHERTY TJ. (1987). Photosensitizers: therapy and detection of malignant tumours. *Photochem. Photobiol.*, **45**, 879–889.

DOUGHERTY TJ. (1993). Photodynamic therapy. *Photochem. Photobiol.*, **58**, 895–900.

EVENSEN JF, SOMMER S, RIMINGTON C AND MOAN J. (1987). Photodynamic therapy of C3H mammary carcinoma with haematoporphyrin diethers as sensitisers. *Br. J. Cancer*, **55**, 483–486.

FORD WE, RIHTER BD, KENNEY ME AND RODGERS MAJ. (1989). Photoproperties of alkoxy-substituted phthalocyanine with deep red optical absorbance. *Photochem. Photobiol.*, **50**, 277–282.

HENDERSON BW AND BELLNIER DA. (1989). Tissue localization of photosensitizers and the mechanism of photodynamic tissue destruction. In *Ciba Foundation Symposium 146, Photosensitizing Compounds: Their Chemistry, Biology and Clinical Use*, pp. 112–125. John Wiley: Chichester.

ISELE U, SCHIEWECK K, KESSLER R, VAN HOOGEVEST P AND CAPRARO HG. (1995). Pharmacokinetic and body distribution of liposomal Zinc phthalocyanine in tumour-bearing mice: influence of aggregation state, particle size and composition. *Biochem. Pharmacol.* (submitted).

JORI G. (1987). Photodynamic therapy of solid tumours. *Radiat. Phys. Chem.*, **30**, 375–380.

JORI G. (1989). In vivo transport and pharmacokinetic behaviour of tumour photosensitizers. In *Ciba Foundation Symposium 146, Photosensitizing Compounds: Their Chemistry, Biology and Clinical Use*, pp. 78–94. John Wiley: Chichester.

KONGSHAUG M. (1992). Minireview: distribution of tetrapyrrole photosensitizers among human plasma proteins. *Int. J. Biochem.*, **24**, 1239–1265.

KONGSHAUG M, CHENG LS, MOAN J AND RIMINGTON C. (1991). Interaction of cremophor EL with human plasma. *Int. J. Biochem.*, **23**, 473–478.

KORBELIK M. (1993). Distribution of disulfonated and tetrasulfonated aluminium phthalocyanine between malignant and host cell populations of a murine fibrosarcoma. *J. Photochem. Photobiol. B, Biol.*, **20**, 173–181.

KREMER JMH, ESKER MWJ, PATHMAMANOHARAN C AND WIERSEMA PH. (1977). Vesicles of variable diameter prepared by a modified injection method. *Biochemistry*, **16**, 3932–3935.

LOMBARDI P, NORATA G, MAGGI FM, CANTI G, FRANCO P, NICOLIN A AND CATAPANO AL. (1989). Assimilation of LDL by experimental tumours in mice. *Biochim. Biophys. Acta*, **1003**, 301–306.

MAZIÈRE JC, MOLIÈRE P AND SANTUS R. (1991). The role of the low density lipoprotein receptor pathway in the delivery of lipophilic photosensitizers in the photodynamic therapy of tumours. *J. Photochem. Photobiol. B, Biol.*, **8**, 351–360.

MILANESI C, BIOLO R, REDDI E AND JORI G. (1987). Ultrastructural studies on the mechanism of the photodynamic therapy of tumours. *Photochem. Photobiol.*, **46**, 675–681.

MOAN J AND BERG K. (1992). Photochemotherapy of cancer: experimental research. *Photochem. Photobiol.*, **55**, 931–948.

MORGAN AR, GARBO GM, KREIMER-BIRNBAUM M, KECK RW, CHAUDHURI K AND SELMAN SH. (1987). Morphological study of the combined effect of purpurin derivatives and light on transplanted bladder tumours. *Cancer Res.*, **47**, 496–498.

POLO L, REDDI E, GARBO GM, MORGAN AR AND JORI G. (1992). The distribution of the tumour photosensitizers Zn(II)-phthalocyanine and Sn(IV)-etiopurpurin among rabbit plasma proteins. *Cancer Lett.*, **66**, 217–223.

PRATESI R. (1984). Diode lasers in photomedicine. *IEEE J. Quant. Electr.*, **20**, 1433–1439.

REDDI E, ZHOU C, BIOLO R, MENEGALDO E AND JORI G. (1990). Liposome- or LDL-administered Zn(II)-phthalocyanine as a photodynamic agent for tumours. I. Pharmacokinetic properties and phototherapeutic efficiency. *Br. J. Cancer*, **61**, 407–411.

RIHTER BD, KENNEY ME, FORD WE AND RODGERS MAJ. (1990). Synthesis and photoproperties of diamagnetic octabutoxy-phthalocyanines with deep-red optical absorbance. *J. Am. Chem. Soc.*, **112**, 8064–8070.

SCHERPHOF GL, SPANJER HH, DERKSEN JTP, LAZAR G AND ROERDINK FH. (1989). Targeting of liposomes to liver cells. In *Drug Carrier Systems*, Roerdine FH and Kroon AM. (eds) pp. 281–291. John Wiley: Chichester.

SEGALLA A, MILANESI C, JORI G, CAPRARO HG, ISELE U AND SCHIEWECK K. (1994). CGP 55398, a liposomal Ge(IV)phthalocyanine bearing two axially ligated cholesterol moieties: a new potential agent for photodynamic therapy of tumours. *Br. J. Cancer*, **69**, 817–825.

SOUNIK JR, SCHECHTMAN LA, RIHTER BD, FORD WE, RODGERS MAJ AND KENNEY ME. (1990). Synthesis and characterization of naphthalocyanines and phthalocyanines of use in sensitizer studies. In *Photodynamic Therapy: Mechanisms II*, Vol. 1203. Dougherty TJ (ed.) pp. 224–232, SPIE Proceedings Series: Bellingham.

TERPSTRA AHM, WOODWARD CJH AND SANCHEZ-MUNIZ FJ. (1981). Improved techniques for the separation of serum lipoproteins by density gradient ultracentrifugation: visualization by prestaining and rapid separation of serum lipoproteins from small volumes of serum. *Anal. Biochem.*, **111**, 149–157.

TRALAU CJ, BARR H, SANDEMAN DR, BARTON T, LEWIN NR AND BOWN SG. (1987). Aluminum sulfonated phthalocyanine distribution in rodent tumours of the colon, brain and pancreas. *Photochem. Photobiol.*, **46**, 777–781.